# Opportunities and Challenges for Using Automatic Human Affect Analysis in Consumer Research

**DOI:** 10.3389/fnins.2020.00400

**Published:** 2020-04-28

**Authors:** Dennis Küster, Eva G. Krumhuber, Lars Steinert, Anuj Ahuja, Marc Baker, Tanja Schultz

**Affiliations:** ^1^Cognitive Systems Lab, Department of Mathematics and Computer Science, University of Bremen, Bremen, Germany; ^2^Department of Psychology and Methods, Jacobs University Bremen, Bremen, Germany; ^3^Department of Experimental Psychology, University College London, London, United Kingdom; ^4^Maharaja Surajmal Institute of Technology, Guru Gobind Singh Indraprastha University, New Delhi, India; ^5^Centre for Situated Action and Communication, Department of Psychology, University of Portsmouth, Portsmouth, United Kingdom

**Keywords:** automatic human affect analysis (AHAA), machine learning, facial expression, spontaneous expressions, dynamic responses, consumer research

## Abstract

The ability to automatically assess emotional responses via contact-free video recording taps into a rapidly growing market aimed at predicting consumer choices. If consumer attention and engagement are measurable in a reliable and accessible manner, relevant marketing decisions could be informed by objective data. Although significant advances have been made in automatic affect recognition, several practical and theoretical issues remain largely unresolved. These concern the lack of cross-system validation, a historical emphasis of posed over spontaneous expressions, as well as more fundamental issues regarding the weak association between subjective experience and facial expressions. To address these limitations, the present paper argues that extant commercial and free facial expression classifiers should be rigorously validated in cross-system research. Furthermore, academics and practitioners must better leverage fine-grained emotional response dynamics, with stronger emphasis on understanding naturally occurring spontaneous expressions, and in naturalistic choice settings. We posit that applied consumer research might be better situated to examine facial behavior in socio-emotional contexts rather than decontextualized, laboratory studies, and highlight how AHAA can be successfully employed in this context. Also, facial activity should be considered less as a single outcome variable, and more as a starting point for further analyses. Implications of this approach and potential obstacles that need to be overcome are discussed within the context of consumer research.

## Introduction

Emotions matter profoundly for understanding consumers’ behavior in fast changing economic markets of modern life (McStay, 2016). While there exist various ways to assess emotions in the laboratory, most approaches that target bodily signals require sensors to be attached to the participant that are either less accurate or less practicable when used in the field (Küster and Kappas, 2013). Hence, automated methods of measuring facial emotional responses via contact-free video recording tap into a rapidly growing market that presents opportunities but also risks (e.g., Gupta, 2018; Schwartz, 2019), and debate about false expectations (Vincent, 2019).

If consumer attention, social engagement, and emotional responses can be measured reliably and non-invasively, a broad spectrum of marketing decisions could be readily informed by objective data. As such, we need to examine *how well* new computational methods can predict consumer behavior, thereby moving away from questions that simply ask *whether* or not they can predict choice (Smidts et al., 2014). Further, it will be critical to measure neurocognitive choice processes in more naturalistic settings to facilitate the study of a broad spectrum of human behavior – including also disorders such as addiction and obesity (Hsu and Yoon, 2015). For example, such settings could include the elicitation of complex yet distinct mixed emotional states such as the feeling of *being moved* that is often described as pleasurable, but that can also involve crying and tears (Zickfeld et al., 2019). Viewing another’s tears has been shown to elicit empathy and a wish to help (e.g., Küster, 2018). In turn, this might result in increased donations towards advertisements based on the feeling of being moved. Potentially, it might be possible to even simulate human-like empathy through affective computing (Picard, 1997), thereby creating an “empathic artificial intelligence” (McStay, 2018) that fundamentally transforms the future of consumer research and related fields. On the flipside, certain real-world applications of automatic human affect analysis (AHAA), such as the detection of unhappy emotional states of customers in retail stores (e.g., Anderson, 2017) appear to be vastly premature, if not downright unethical.

The current paper aims to critically discuss the growing role of AHAA in consumer research. It also highlights some of the most pressing barriers the field currently faces. We argue that automatic classification may provide substantial new leverage to the study of emotion and cognition in consumer neuroscience through both primary and subsequent machine analysis. While the tools available to date may not be as versatile, reliable, and proven to be valid across domains, they nevertheless represent an important advance in the area of AHAA with substantial potential for further development.

## Abundant Choices: Classifiers Lack Cross-System Validation

In the past decades, early automated systems for facial affect recognition (Tian et al., 2001) were not readily available for use by the wider research community. In the wake of recent technical advances in video-based affect sensing, this has changed (Valstar et al., 2012). Today, researchers face a plethora of choices for selecting the best machine classifier. Besides covering a wide range of price tags, commercial systems differ in their technical features for facial analysis, as well as the ways in which users engage with the system (e.g., through APIs, SDKs). Among the “grizzled veterans” in the field of AHAA are the two software packages FACET (iMotions) and FaceReader (Noldus). Originally built upon another software called CERT (Littlewort et al., 2011), FACET was distributed by Emotient, whereas FaceReader was developed and first presented by VicarVision in 2005 (Den Uyl and Van Kuilenburg, 2005). Both systems have been used in a large number of scientific studies (e.g., Skiendziel et al., 2019; for a review see Lewinski et al., 2014a) as well as in consumer behavior (Garcia-Burgos and Zamora, 2013; Danner et al., 2014; Yu and Ko, 2017) and marketing research (Lewinski et al., 2014b; McDuff et al., 2015). Nonetheless, there are several other promising off-the-shelf classifiers available today that could be employed for the same purposes. These include *Affdex* (Affectiva), *FaceVideo* (CrowdEmotion), *Cognitive Services: Face* (Microsoft), *EmotionalTracking* (MorphCast), *EmotionRecognition* (Neurodata Lab), or *FaceAnalysis* (Visage Technologies). Moreover, some classifiers are freely available such as OpenFace (Baltrusaitis et al., 2016) or OpenCV (Bradski, 2000) to extract facial feature sets from video recordings.

Given the large and growing number of choices for academics and practitioners in consumer research, there still exists little “cross-system” (i.e., between competing products) validation research that could independently inform about the relative performance indicators of AHAA (Krumhuber et al., 2019). Out of the studies available to date, only a few have directly compared different commercial classifiers (Stöckli et al., 2018). Likewise, a small number of studies has tested AHAA against human performance benchmarks on a larger number of databases (Yitzhak et al., 2017; Krumhuber et al., 2019), thereby calling the generalizability of findings derived from single stimulus sets into question.

Ultimately, not only accuracy of AHAA needs to be evaluated, but also its validity and reliability in a broader sense (cf., Meyer, 2010; Ramsøy, 2019). First, certain concepts may require re-interpretation: For example, classic *test–retest reliability* by the machine classifier on identical stimuli tends to be perfect because the underlying algorithms remain fixed. Likewise, the issue of *inter-rater reliability*, i.e., different experimenters applying the same AHAA, may be irrelevant if all parameters are shared between experimenters. More critical, however, are questions of *convergent* and *external validity*. So far, most validation efforts have focused on the convergence between AHAA and human ratings, although initial evidence suggests that AHAA may correlate highly with facial electromyography (EMG; Kappas et al., 2016; Beringer et al., 2019; Kulke et al., 2020). However, much more work is still needed to compare AHAA against both facial EMG and expert annotations to determine its *convergent* and *discriminant validity*. *Generalizability* of AHAA study findings may be further limited in other ways. E.g., classifier performance may be substantially lower for spontaneous affective behavior (Dupré et al., 2019; Krumhuber et al., 2019). This issue often ties into the lack of information given about the stimulus materials originally used to develop or “train” a given AHAA system to fully evaluate generalizability toward similar novel stimuli.

Finally, AHAA needs to demonstrate an added value to predict consumer behavior. A few studies have begun to examine this question by predicting purchase intent from automatically detected facial expressions. For example, the FACET classifier has recently been employed to examine purchase intent toward vegetable juices, showing that AHAA-based facial expressions modulated consumer intent in concert with a number of other factors (Samant et al., 2017; Samant and Seo, 2020). Nevertheless, it remains an empirical question to what extent expressions, as tracked by AHAA, translate to purchase intent and tangible real-world behavior.

Overall, we still know too little about the various contenders to choose between classifiers for different purposes. Once a commercial software package has been purchased, users typically have few options to reconsider their choice, as the cost of even a single system is often in the (higher) four- or (lower) five-digit range. Furthermore, available open-source solutions such as *OpenFace* still need to be tested with regards to their potential for supplementary behavioral analysis.

## Missing the Beat of Fine-Grained Expression Dynamics

In the real world, faces are constantly in motion. As demonstrated by a growing body of research in cognitive science, the dynamics of facial movement convey communicative intent and emotion (for reviews, see Krumhuber et al., 2013; Krumhuber and Skora, 2016; Sato et al., 2019). While the role of fine-grained dynamics has been best explored in the context of smiling (e.g., Krumhuber et al., 2007, 2009), they are believed to impact emotion judgments and behavioral responses more generally (Sato and Yoshikawa, 2007; Recio et al., 2013). This renders expression dynamics to be of crucial importance for large areas of consumer research. Since online and TV advertisements frequently involve dynamic material involving human faces, their affective credibility depends on whether the content is perceived as genuine-looking and sincere. However, relatively little is known about the precise characteristics of expression dynamics in product evaluation beyond simple analyses of means (Peace et al., 2006). Teixeira and Stipp (2013) showed an inverted-U relationship between smile intensity and purchase intents of people who viewed advertisements – i.e., both very high and very low levels of humorous entertainment predicted lower purchase intent. Similarly, *joy velocity*, i.e., the speed of change in facial expressions of happiness, has been suggested to affect consumers’ decisions to continue to watch or “zap” advertisements (Teixeira et al., 2012). Finally, humorous entertainment, as measured by smile intensity, may increase purchase intent when placed after, rather than before, brand presentation (Teixeira et al., 2014).

One reason for this comparative neglect of the dynamics of facial movement lies in its complexity. In traditional laboratory research, a limited number of factors can be manipulated simultaneously. Higher ecological validity of evoked facial activity, and more natural recording situations, typically make it more difficult to adequately control for possible confounds, as well as ensure sufficient statistical power. As shown in prior research (Ambadar et al., 2005), the impact of dynamic expressions is likely to be more than the sum of still images. While temporal information improves emotion recognition (Krumhuber et al., 2013), it is less clear how multi-peaked dynamic expression trajectories are weighted in the mind of the human perceiver. Also, it remains largely unknown how rich socio-emotional knowledge about the context of dynamic expressions shapes their perception (Maringer et al., 2011). Such applied questions are of imminent relevance for consumer research given that AHAA can provide per-frame classifications of large amounts of video data of human observers. For example, based on an analysis of more than 120,000 frames, Lewinski et al. (2014b) found context-specific features of facial expressions of happiness to be major indicators of happiness. Unfortunately, however, no well-established standards yet exist in terms of how best to pre-process and aggregate raw per-frame probabilities of machine classification.

Until now, many validation approaches consider only the peak response intensities or overall mean response envelopes of a perceiver’s facial activity. From our perspective, this calls for more advanced and systematic ways of generating and testing hypotheses relating to short- to medium term expression dynamics. Such challenges may require the use of metrics that do not simply reduce complex facial movements to a single image, i.e., one that is representative of the prototypical peak expression. Instead, temporal segments of facial activity need to be weighted relative to other simultaneously present channels, without discarding nuanced expressions (Pantic and Patras, 2006; Valstar and Pantic, 2006; Dente et al., 2017).

While AHAA provides new avenues for more fine-grained and subtle expression analysis, certain use cases might fail to translate to future research. For example, it is unlikely that micro-expressions (Ekman, 2009; Matsumoto and Hwang, 2011) offer a promising theoretical approach toward a better understanding of expression dynamics in consumer research. Micro-expressions refer to brief displays (20–500 ms) argued to “leak” an individual’s true emotional state before the expression can be actively controlled (Ekman and Friesen, 1969). While micro-expression analysis still enjoys substantial attention (see Shen et al., 2019), the concept is questionable and lacks empirical support as a validated theory, partly because micro-expressions are extremely rare (Porter et al., 2012) and of little practical relevance in understanding the multiple functions of emotions. As such, they could simply represent briefer and weaker versions of normal emotional expressions (Durán et al., 2017). In consequence, it seems worthwhile to focus future research efforts on other aspects, such as those that concern dynamic and spontaneous emotional behavior beyond the level of the individual frame.

## From Posed Stereotypes to Spontaneous Expressions “In the Wild”

Video-based affect classification can only be a useful tool for consumer research if patterns of naturally occurring responses can be reliably detected. Historically, AHAA has primarily been designed to achieve high accuracy in recognizing intense and stereotypical expressions provided by carefully instructed actors (Pantic and Bartlett, 2007). However, the narrow focus on individual posed emotions throughout psychology has been increasingly criticized because it has not been very helpful to understand the evolutionary functions of emotional expressions themselves (Shariff and Tracy, 2011). While promising methods for analyzing spontaneous behavior have been proposed, fewer efforts target the automatic analysis of spontaneous displays (Masip et al., 2014). This could be due to the rather limited number of available databases with naturalistic and spontaneous expressions used to train and test machine classifiers. Often, these databases are also of lower recording quality which hinders objective measurement and analysis (Krumhuber et al., 2017).

Recent findings regarding the classification performance of posed expressions have been encouraging. For example, Stöckli et al. (2018) demonstrated acceptable accuracy in classifying basic emotions using the software packages FACET and Affdex. The authors calculated recognition accuracy for maximum intensity expressions extracted from two posed databases. However, when participants were asked to spontaneously respond to emotionally evocative pictures, accuracy for emotional valence (see Yik et al., 2011) was barely above chance level. Similar results have been reported by Yitzhak et al. (2017) using videos. Depending on the emotion in question, recognition performance of prototypical posed expressions typically ranged between 70 and 90%, with happiness being recognized most accurately. By contrast, the same classifier performed “very poorly” (Yitzhak et al., 2017, p. 1) on subtle and non-prototypical expressions. Overall, machine learning for spontaneous expressions is a difficult task, with performance rates varying as a function of classifier, emotion, and database (Dupré et al., 2019). Furthermore, the notion of what constitutes spontaneous facial behavior varies between the databases.

To make significant progress in the future, more work is still needed to create and validate large and diverse datasets of spontaneous expressions (Zeng et al., 2009). For example, efforts such as AffectNet (Mollahosseini et al., 2019) or Aff-Wild (Kollias et al., 2019) might help to close the gap toward predicting affective responses in the wild. Ideally, new databases should be publicly accessible to allow for independent verification of the results or modification of the computer models. Dedicated large scale efforts to obtain high quality “in-the-wild” dynamic facial response data will allow researchers to consistently address ethical challenges that require substantial consideration. E.g., the partial deception required to ensure unbiased responses can be ameliorated through standardized debriefing procedures. Further, spontaneous databases can be (re-)used for multiple cross-system validation studies, as well as for more specific consumer response analyses. By doing so, AHAA of spontaneous expressions may contribute to increasingly better predictions of real-world consumer responses while minimizing the burden on ethical data collection in the field. Finally, such an approach would also provide a benchmark for comparisons between the different algorithms. For example, although a large amount of online video data used for the training of *Affdex* has been one its major selling points (Zijderveld, 2017), this and similar systems still function like a “black box” that cannot be directly validated by other parties.

## Theoretical Issues: a Lack of Coherence

While some of the most pressing issues of AHAA concern practical limitations, theoretical issues equally need to be addressed. Importantly, the notion of a direct and hard-wired or “universal” link between facial expressions and subjective experience has been challenged in recent years. As argued by multiple researchers (Reisenzein et al., 2013; Hollenstein and Lanteigne, 2014; Durán et al., 2017), coherence between emotions and facial expressions may at best be moderate in strength, and sometimes even non-existent. Further, similar configurations of facial actions [i.e., Action Units (AUs)] may express more than one emotion or communicative intent (Barrett et al., 2019). This contrasts with existing views such as those proposed by Basic Emotion Theory (Ekman, 1992, 1999). In consequence, any facial activity, whether it is measured manually or automatically, cannot be assumed to directly reflect a person’s emotional experience. Facial expressions are *not* the sole readout of underlying emotional states (Kappas, 2003; Crivelli and Fridlund, 2018). As a result, AHAA is essentially about the recognition of patterns and regularities in the data (Mehta et al., 2018).

Nevertheless, there are reasons to be optimistic when attempting to interpret facial expressions. First, spontaneous consumer responses might be more predictive of affective behavior than abstract and decontextualized situations as typically examined in the laboratory (Küster and Kappas, 2014). Such applied contexts could be more informative about the emotional experience of respondents, thereby increasing the magnitude and coherence of the response. Second, recent improvements in efficiency rendered by AHAA allows the processing of larger amounts of data than has previously been possible. This should increase overall robustness in study findings across domains, including larger-scale studies (Garcia et al., 2016). Third, results obtained via frame-based classification could be used as a starting point for further analyses based on machine learning despite low overall levels of emotion-expression coherence. For example, for the prediction of consumption choices between several products, it might not matter whether a given smile or frown reflects a full-blown emotion or something else (i.e., concentration, politeness) – provided the consumer’s decision is predicted correctly.

Overall, we therefore propose to consider the wider context of emotional expressions rather than to limit investigations to a blind use of emotion labels provided by commercial machine classifiers without considering the wider context. Instead, it is commendable to think of these technologies as a means to “pre-process” large amounts of facial activity data at low cost. These pre-processed facial activity data can then itself be used as input features for machine learning methods to learn and predict human emotional behavior in context.

## Conclusion

AHAA promises to revolutionize research in consumer neuroscience. However, even apart from general theoretical limitations, its validity and usefulness are likely to vary between different types of studies. Testing hypotheses about specific consumer responses may often depend on relatively small datasets of facial responses, rendering the decision of which software to use even more difficult. In many cases, freely available tools such as OpenFace may be a good entry point. However, there presently appears to be no single software tool on the market that clearly outperforms all other machine classifiers. Hence, additional research is still needed to examine the reliability and predictive value of AHAA. Although the future of automatic affect sensing in consumer research looks promising, it is important to remain aware of its potential limitations. Social scientists can play an active role here to contribute to further development of this technology.

## Author Contributions

DK and EK developed the theoretical ideas. All authors contributed to the discussion and refinement of the presented perspective with regards to AHAA. DK and EK wrote the manuscript. MB and TS provided critical feedback. DK, EK, MB, and LS contributed to manuscript revision. All authors read and approved the final version.

## Conflict of Interest

The authors declare that the research was conducted in the absence of any commercial or financial relationships that could be construed as a potential conflict of interest.
